# Mitochondrial Targeting Therapy Role in Liver Transplant Preservation Lines: Mechanism and Therapeutic Strategies

**DOI:** 10.7759/cureus.16599

**Published:** 2021-07-23

**Authors:** Anjli Tara, Jerry Lorren Dominic, Jaimin N Patel, Ishan Garg, Jimin Yeon, Marrium S Memon, Sanjay Rao Gergal Gopalkrishna Rao, Seif Bugazia, Tamil Poonkuil Mozhi Dhandapani, Amudhan Kannan, Ketan Kantamaneni, Myat Win, Terry R Went, Vijaya Lakshmi Yanamala, Jihan A Mostafa

**Affiliations:** 1 General Surgery, California Institute of Behavioral Neurosciences & Psychology (CIBNP), Fairfield, USA; 2 General Surgery, Liaquat University of Medical and Health Sciences (LUMHS), Jamshoro, PAK; 3 General Surgery, Vinayaka Mission's Kirupananda Variyar Medical College, Salem, IND; 4 General Surgery, Stony Brook Southampton Hospital, New York, USA; 5 General Surgery and Orthopaedic Surgery, Cornerstone Regional Hospital, Edinburg, USA; 6 Family Medicine, California Institute of Behavioral Neurosciences & Psychology (CIBNP), Fairfield, USA; 7 Medicine, California Institute of Behavioral Neurosciences & Psychology (CIBNP), Fairfield, USA; 8 Research, California Institute of Behavioral Neurosciences & Psychology (CIBNP), Fairfield, USA; 9 Internal Medicine, California Institute of Behavioral Neurosciences & Psychology (CIBNP), Fairfield, USA; 10 Faculty of Medicine, California Institute of Behavioral Neurosciences & Psychology (CIBNP), Fairfield, USA; 11 Internal Medicine/Family Medicine, California Institute of Behavioral Neuroscience & Pyshology (CIBNP), Fairfield, USA; 12 Internal Medicine, Medical City Plano, Plano, USA; 13 Medicine, Jawaharlal Institute of Postgraduate Medical Education and Research (JIPMER), Puducherry, IND; 14 General Surgery Research, California Institute of Behavioral Neurosciences & Psychology (CIBNP), Fairfield, USA; 15 Surgery, California Institute of Behavioral Neurosciences & Psychology (CIBNP), Fairfield, USA; 16 Surgery, Dr.Pinnamaneni Siddhartha Institute of Medical Sciences and Research Foundation, Gannavaram, IND; 17 General Surgery, Nottingham University Hospitals NHS Trust, Nottingham, GBR; 18 Psychiatry and Behavioral Sciences, California Institute of Behavioral Neurosciences & Psychology (CIBNP), Fairfield, USA

**Keywords:** liver transplantation, mitochondrial targeting therapy, ischemia/reperfusion injury, mechanism, preservation, therapeutics, graft survival, graft failure, pathophysiology

## Abstract

The normal function of mitochondria in the hepatic parenchyma can be disrupted by ischemia/reperfusion (I/R) damage during liver transplantation. The pathology of these insults involves various cellular and molecular steps of events that have been extensively researched over decades but are yet to provide complete answers. This review discusses the brief mechanism of the pathophysiology following ischemia/reperfusion injury (IRI) and various targeting strategies that could result in improved graft function.

The traditional treatment for end-stage liver disease i.e., liver transplantation, has been complicated by I/R damage. The poor graft function or primary non-function found after liver transplantation may be due to mitochondrial dysfunction following IRI. As a result, determining the sequence of incidents that cause human hepatic mitochondrial dysfunction is crucial; it might contribute to further improvements in the outcome of liver transplantation. Early discovery of novel prognostic factors involved in IRI could serve as a primary endpoint for predicting the outcome of liver grafts as well as promoting the early implementation of novel IRI-prevention strategies. In this review, recent developments in the study of mitochondrial dysfunction and I/R damage are discussed, specifically those concerning liver transplantation. Furthermore, we also explore different pharmacological therapeutic methods that may be used and their connections to mitochondrion-related processes and goals.

Although significant progress has been made in our understanding of IRI and mitochondrial dysfunction, further research is needed to elucidate the cellular and molecular pathways underlying these processes to help identify biomarkers that can aid donor organ evaluation.

## Introduction and background

Don't think of organ donations as giving up part of yourself to keep a total stranger alive. It's a total stranger giving up almost all of themselves to keep part of you alive. - Aksh Dhiman [1]

Liver transplantation (LT) is the treatment of choice for end-stage liver disease and irreversible tumors of hepatic origin [2]. The first human liver transplant was performed by Thomas Starzl in 1963. Today, with the help of technology and effective immunosuppressive therapies, the five-year survival of patients undergoing LT has increased up to 75% [3]. The application of the LT procedure is limited by significant organ availability. This scarcity has led to the consideration of extended criteria donor (ECD) grafts, which were previously considered unfit for transplantation i.e., the inclusion of older donors, donation after hepatic steatosis and cardiac death, organs after prolonged cold ischemia time (CIT) [4,5].

**Figure 1:**
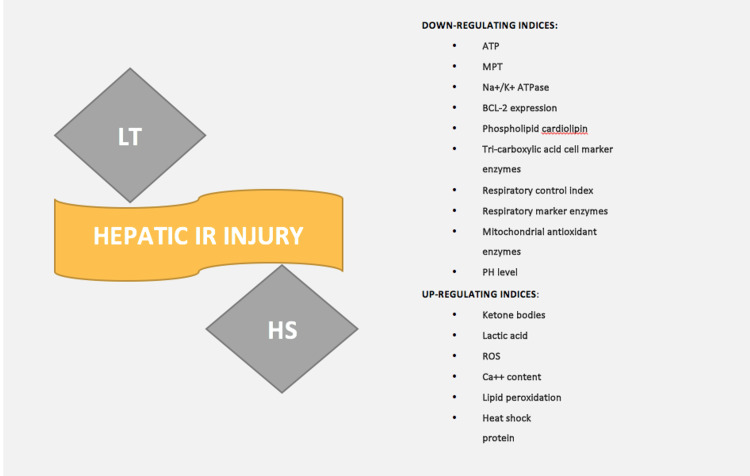
LT – Liver transplantation, HS – Hemorrhagic shock. Hepatic IRI can be classified into cold I/R (LT-induced) and warm I/R (e.g. HS-induced); the up-regulating and down-regulating indices are given in the above figure.

The liver is the largest organ with many synthetic and secretion functions. Due to its maximum exposure to the outside environment, the liver is most susceptible to ischemia/reperfusion injury (IRI). IRI is a multifactorial pathological complication that may result in parenchymal cell damage/death in an organ that is initiated primarily by decreased oxygen availability or hypoxia, which is then aggravated once oxygen and pH are restored [4]. This is the major phenomenon that leads to the initiation of parenchymal injury in major surgeries like LT. I/R is mainly classified into two types: warm and cold. Warm IRI mainly occurs secondary to decreased blood flow [6,7], whereas cold IRI is exclusively coupled with LT. In cold IRI, the donor graft is preserved in static cold storage (SCS) before warm reperfusion [8]. The demonstration of the up-regulating and down-regulating indices following hepatic IRI is shown in Figure 1.

Even after the current advances in surgical technology, primary graft failure, and graft rejection still occur unforeseeably. And so, it is very important to understand the mechanical and molecular background behind this pathology to make further medical advancements.

The most fundamental event that results in IRI is the mitochondrion functional deterioration or mitochondrial permeability transition (MPT) [9,10]. This change leads to conversion of aerobic metabolism to anaerobic, consequently resulting in the dysfunction of adenosine triphosphate (ATP)-producing mitochondrial enzymes. The decreased energy level in the stressed parenchymal cells leads to the initiation of necrosis by the release of numerous inflammatory cytokines and chemokines, which in turn divert circulating polymorphonuclear leukocytes to reperfused tissue causing additional injury. The damaged mitochondrion cells also encounter decreased expression of B-cell lymphoma 2 (BCL-2). Cytochrome c then leaks and activates caspase cascade and promotes apoptosis. Simultaneously, the ischemic cell also suffers calcium overload that causes lipid peroxidation and promotes further membrane damage. The reactive oxygen species (ROS) also causes significant damage to the parenchymal cells by the action of oxidases. Additionally, upon reperfusion, the damaged mitochondria release damage-associated molecular patterns (DAMPs) that lead to organ dysfunction and rejection by activation of the innate immune response, which then causes inflammatory damage to the tissue [11]. A number of pre-treatments that can reduce damage following hepatic injury are currently being investigated. These include ischemic preconditioning (IPC), pre-treatments involving non-physiological oxygen levels, pharmaceutical preconditioning, and gene targeting methods [12].

In this review, we will be collecting information from previously published articles and will be examining the subsequent mechanism, pathology, and strategy for targeting therapeutic interventions aimed at preserving mitochondrial function that could be used to minimize hepatic IRI. As mitochondria are widely distributed in liver tissue, we need to understand how mitochondrial dysfunction affects hepatic I/R-induced injury, thus also revealing novel therapeutic approaches for treating hepatic I/R.

## Review

Liver transplantation

Liver transplantation is the definitive procedure for end-stage liver disease; the most common indications are hepatocellular carcinoma, alcoholic liver disease, and hepatitis C virus infection. Other indications that are applicable are cholestatic liver disease, metabolic disorders, and acute fulminant liver failure secondary to various causes. The LT procedure has developed immensely from being experimental to having great clinical significance over the years. Advancements in surgical techniques, donor selection, postoperative care, and immunosuppressive regimen have led to the long-term survival of the graft. Notably, the hazard of most important graft non-function after the transplant of fatty donor organs is markedly greater than that of non-steatotic grafts (60% vs. 5%). Severe macrovesicular steatosis (> 60%) has been linked with > 60% risk of the main non-function after transplantation, and this has been calculated to be responsible for the rejection of 25% of donor livers [13]. In addition, the number of patients on the waiting list for transplants has been increasing immensely with the consequent shortage of donors. To address this concern, the model for end-stage liver disease (MELD) score was endorsed in 2002. This score is used globally to select patients in the recipient list by predicting their three-month mortality from the existing liver disease that ought to receive specific donor organs.

Organ Preservation Techniques

The net consequence of the LT is potentially affected by the preservation, an important step in LT. The gold standard preservation technique for organ transplantation is simple/static cold storage (SCS), which has been used widely and was first introduced by Geoffrey Collins in 1969. This technique enlists the use of standard preservation solutions such as the University of Wisconsin (UW) solution, histidine-tryptophan-ketoglutarate (HTK), and Celsior solutions on ice, at low temperatures (2°C to 4 °C) for liver graft preservation [14]. Despite the benefits from this standard technique, tissue injury is still an issue especially in the ECD grafts or extended CIT. Techniques like hypothermic machine perfusion (HMP) have been evolved, where the graft is continuously feeding on preservative solutions in order to avoid undesirable consequences. The continuous supply of nutrients and constant removal of toxic metabolites gives this technique an edge over the SCS. The complexity and high expense of HMP though makes it a less favorable approach than SCS. However, to avoid cold ischemic injuries to the graft, scientists have developed new techniques to preserve transplantation organs at higher or more physiologically friendly temperatures. The current strategies used for perfusion of the graft are erythrocyte-based solutions, oxygenated autologous blood, or a cellular solution at normothermic temperatures (35°C to 38°C) for normothermic machine perfusion (NMP) or, sub-normothermic temperatures (25°C to 34°C) for sub-normothermic machine perfusion (SMP) [15]. Although issues with NMP are various including its demand for specialized intricacy and its lack of financial feasibility, it has been shown to improve outcomes in terms of lower early allograft dysfunction (EAD) and aspartate transaminase (AST) levels in contrast to SCS [16]. The most recent preservation techniques such as ex vivo machine perfusion are presently under research. Other diverse temperatures (hypothermia or normothermia), and multiple preservative solutions are also under consideration. The in-depth examination and growth of new strategies will most likely result in an alteration in the methods by which organs are perfused, preserved, and transported.

Ischemia/reperfusion (I/R) injury

**Figure 2:**
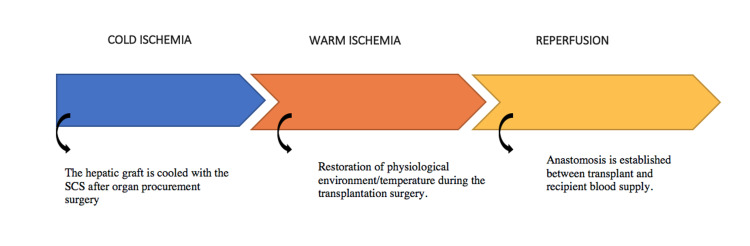
SCS - Static cold storage. Schematic representation of different phases of the hepatic ischemic reperfusion injury.

I/R injury (IRI) is the pathological process that is the most common cause of organ transplant rejection/failure. It results initially in ischemia while stored at cold temperatures, and exacerbates with the return to physiological temperature after transplant and when connected to the recipient blood. This subject is under intense research over the last decade, but due to the complexity of this pathology, it has yet to be fully unlocked. The different phases of hepatic I/R have been identified in Figure 2.

The first phase results in ischemia due to functional changes while under cold preservation. In contrast, the second phase is the exaggeration of the initial insult, after when the graft undergoes reperfusion [17]. The presentation of the second phase is again divided into two, namely early-stage and late-stage. Early-stage occurs within two hours of reperfusion and is mostly driven by kupffer cells (KCs), sinusoidal endothelial cells (SECs), and the resultant ROS generation. Late-stage occurs in six to 48 hours after reperfusion and is driven by infiltration of the immune cells such as neutrophils, or cluster differentiation 4+ (CD4+) T-lymphocytes that accelerate cell degeneration by releasing cytotoxic enzymes. Hepatic IRI in some cases can result in multi-organ dysfunction syndrome (MODS) or systemic inflammatory response syndrome (SIRS), which have been associated with high rates of mortality and morbidity [18]. The key mediators and factors included in the pathophysiology of hepatic IRI are oxidative stress, anaerobic metabolism, nitrous oxide (NO), KCs and neutrophils, mitochondria, intracellular calcium overload, cytokines, and chemokines.

Mitochondria Dysfunction Following IRI

Initial ischemia results in the decreased oxidative phosphorylation i.e. decreased production of ATP that leads to the initiation of anaerobic respiration followed by increased concentration of hydrogen (H+) ion, generating acidosis in the body. This event occurs secondary to when the graft is stored in a cold preservative. The H+ concentration inside the cell leads to the dysfunction of the sodium-hydrogen (Na+/H+) exchanger that results in the accumulation of sodium (Na+) inside the cell. Also, decreased activity of sodium-potassium adenosine triphosphatase (Na+/K+ ATPase) pump leads to further exacerbation of Na+ accumulation that results in cell swelling and death. This disruption also results in increased intracellular calcium concentration via impaired homeostasis of the sodium-calcium (Na+/Ca++) exchanger due to prevalent ischemia [19]. Upon implantation and reperfusion of the graft, mitochondria contribute to IRI. The intracellular increase in the Na+, calcium (Ca+), H+ ions, contributes to the mitochondrial disruption that leads to the formation of mitochondria permeability transition pores (MPTP), which immediately commence apoptosis and necrosis simultaneously, causing maximum damage [20]. The generation of ROS also contributes to the extensive cellular damage in the IRI following lipid peroxidation, DNA damage, and protein oxidation. Other mediators like neutrophils and KCs also play a key role in the pathogenesis of the IRI by generating ROS, and different chemokines and cytokines such as interleukin-12 (IL-12), interleukin-23 (IL-23), tumor necrosis factor (TNF) alpha, etc. The decreased ratio of nitric oxide (NO) to endothelin also worsens the already prevailing ischemic conditions by promoting the vasoconstriction of the hepatic sinusoids and limiting the flow of blood. NO here has an abundance of importance. It causes vasodilation, inhibits platelet aggregation, inhibits leukocyte adhesion to vascular endothelial cells, and also inhibits caspases to prevent apoptosis. NO is immensely noticeable in regulating innate and adaptive immunity as well as in suppressing pro-inflammatory cascade, hence limiting the damage [21]. IRI is a vast and extensive pathology that is yet to be completely unraveled. The above-mentioned imbalances in the natural homeostasis set off catastrophic events followed by hepatic IRI that lead to primary graft dysfunction and organ rejection. Mitochondria is at the center of all the above-mediated events and hence, should be targeted for future therapeutic strategies wisely for the better survival of the graft and the patient.

Mitochondrial targeting therapies against IRI in liver transplantation

IRI is a multifactorial process. It has shown multiple limitations when studied on animal models and is the reason most of the studies done couldn't be followed up with human trials [11]. Since mitochondrial disruption is the center of the pathophysiology in IRI, many therapeutic targeting strategies in literature have been reported. Multiple experimental investigations suggest various drugs (natural and synthetic) that are promising. However, despite all the reviews, no fixed therapy has been established to date. Many techniques have been currently used to reduce events promoting IRI, such as storage methods (cold preservation, machine perfusion), manual conditioning, and multiple pharmacological conditioning. These indicate an important relationship between mitochondrial activity and mitigation of IRI [22]. Following are instances of mitochondrion-related processes and therapeutic targets to reduce IRI risk.

Mitochondria permeability transition pores (MPTP) initiate cell death by leakage of cytochrome c from the inner mitochondrial membrane, which activates caspases and promotes apoptosis. This phenomenon results secondary to increased intracellular calcium levels. Increased cytosolic Ca++ concentration stimulates Ca++ uni-porters in the mitochondrial membrane that leads to an increase in mitochondrial Ca++ concentration. The disruption of Ca++ homeostasis triggers the formation of MPT pores, which results in cell injury via either apoptosis or necrosis. Pretreatment with calcium channel blockers like amlodipine has proven to reduce the injury risk by normalizing the cellular environment and has also counteracted the mitochondrial disruption induced by hepatic I/R in one study [23]. Another calcium-channel inhibitor - 2-aminoethoxydiphenyl borate (2-APB) - prevented cell death during I/R by decreasing Ca++ overload, and cytochrome c release. In addition, MPT inhibition via cyclosporine A has promoted a significant reduction in ROS generation in response to Ca++.

ROS is responsible for the major deleterious effects in a hepatic IRI after LT. These effects can be reduced by using antioxidants that help with the preservation of ATP production and also increases survival. Mangafodipir trisodium, an antioxidant, has beneficial effects on the prevention of IRI when administrated to the donor before organ harvesting [24]. Also, herbal antioxidants such as green tea catechins, tetrandrine, quercetin, and trans-resveratrol, have the potential to reduce IRI [25]. Furthermore, glutathione is an antioxidant that can contribute a great deal to the survival of the transplanted graft, if targeted. Melatonin, a potent antioxidant, can help in up-regulating the expression of multiple antioxidant enzymes such as superoxide dismutase, glutathione peroxidase, and glutathione reductase [25]; therefore alleviating the risk of hepatic IRI through preservation of ATP synthesis and mitochondrial function. N-acetyl cysteine (NAC), a glutathione precursor, when administrated intravenously, replenishes glutathione stores, improves homeostasis, and increases survival, and hence lowers the incidence of graft dysfunction [26].

Heme oxygenase-1 (HO-1) is an enzyme that mediates the degradation of the heme compound into biliverdin, carbon monoxide (CO), and ferrous ions. HO-1 has been studied to have promising effects on the prevention of hepatic IRI and hence its therapeutic benefits should be considered. Biliverdin scavenges ROS to alleviate the inflammatory events. Ferritin also reduces apoptotic stimulus via its antioxidant function. CO via mitogen-activated protein kinase pathway (MAPK), promotes anti-inflammatory and anti-apoptotic activities that result in microcirculatory maintenance [27]. In addition, HO-1 has also been proved to regulate autophagy in a silent information regulator factor 2-related enzyme 1 (SIRT1)-dependent manner. Activation of SIRT1 is important for the limitation of cell injury against ongoing IRI. The disruption of SIRT1-suppressed autophagy enhancement in mouse liver grafts treated with adenovirus-mediated HO-1 (Ad-HO-1) gene transfer, implies that HO-1 regulates autophagy through SIRT1 [28].

As we know, IRI after LT follows a complex process with ROS generation involving many cellular and molecular pathways. Nicotinamide adenine dinucleotide-dependent deacetylase sirtuin-3 (SIRT3) along with its downstream mediators - superoxide dismutase 2- (SOD2-), cyclophilin D- (CypD-), and hypoxia-inducible factor 1-alpha (HIF-1-alpha) - is responsible for a direct effect on some of the molecular complex events involved in hepatic IRI. It has been explained via recent studies that activation of SIRT3 and its mediators have promising effects on attenuation of the harmful activities of ROS following IRI. And that it may preserve the function of the remaining live tissue following transplantation [29]. Therefore, novel proteins like these should be studied from all possible angles to open further doors to the improvement of the survival of the liver graft.

Several novel targets have been identified with the potential to alleviate the risk of hepatic IRI after LT and promote mitochondrial survival/repair. For example, Akt activators, known as protein kinase B (PKB), belong to a serine/threonine kinase family and play a vital role in cell survival and growth [30]. It is also protective if activated during hepatic IRI. Akt contributes as a key mediator in therapies such as ischemia pre & post-conditioning, micro ribonucleic acid (miRNA)-based therapy, and pharmacological treatments [31]. And so, it needs to be further studied in human trials.

Another instance of a potential treatment is AMP-activated protein kinase (AMPK) activators that perform an essential role in cell energy homeostasis by adjusting the energy metabolism. Therapeutic use of this can promote ATP preservation, alleviate hepatic apoptosis, and hence ease hepatic I/R [30]. In some studies, adiponectin has shown optimistic results in hepatic I/R via AMPK pathway activation favoring this novel strategy in hepatic I/R.

The peroxisome proliferator-activated receptor (PARR) gamma agonist is when the PARR-retinoid X receptor (RXR) forms a heterodimer with the PARR gamma to either induce or repress gene transcription [32]. PARR expression rises in hepatic I/R, increasing hepatocyte resistance to necrosis and apoptosis, and therefore should be considered for human hepatic trial.

The miRNA-based therapies include a class of non-coding RNA molecules that are involved in down-regulating gene expression via different mechanisms. Many studies have demonstrated the success of this targeted therapy in hepatic I/R. For instance, by inhibition, miR-122 has protective effects against hepatic I/R [28]. In addition, RNA interference (RNAi) is a biological mechanism by which RNA molecules suppress gene expression or translation by neutralizing targeted messenger RNA (mRNA) molecules. In RNAi-mediated inhibition of the expression of caspase-8 and caspase-3, the two components of the apoptotic mechanism were suppressed via the intraportal administration of small interfering RNA (siRNAs) that resulted in a reduction in lesions caused in the liver by warm I/R. 

Conditioning of the graft involves two types and is used to decrease the overall risk of IRI. The first is ischemic preconditioning (IPC) where the graft is exposed to brief ischemic stress (five to 10 minutes) via portal triad clamping and is then followed by a longer period of reperfusion (10 to 20 minutes). This reduces the severity of the IRI by attenuating the mitochondrial ROS generation and lowers the oxidative stress significantly. Furthermore, it was noticed in a study that the preconditioned grafts display elevated markers of autophagy, which is one of the cytoprotective mechanisms that promotes longer cell survival. It also increases the HO-1 expression in hepatic stellate cells, endothelial cells, and hepatocytes that are known to promote the prevention of hepatic I/R following LT [33]. During IPC, a mild increase in oxidative stress causes cellular adaptation by reducing ATP synthase activity, thereby preserving hepatic ATP levels and increasing cell tolerance to MPT [34]. IPC also causes an increase in endothelial nitric oxide synthase (eNOS) activity that protects the liver graft against IRI. It has also demonstrated the reduction of alanine aminotransferase (ALT) and aspartate aminotransferase (AST) levels whose markers are a significant representation of liver injury and decreased levels show the protective effects of IPC.

**Figure 3:**
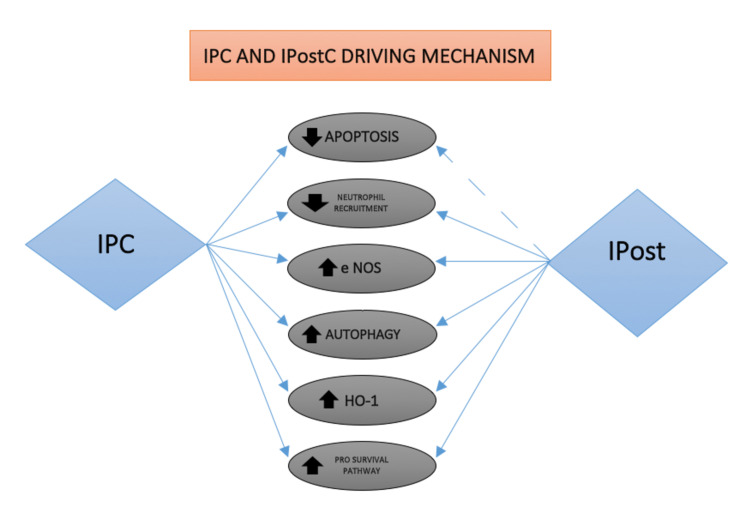
Ischemic preconditioning (IPC) and ischemic post-conditioning (IPostC) mechanisms of action. The mutual mechanisms involved in the operation of IPC and IPostC are depicted in this simplified diagram. In most cases, IPC and IPostC are equally efficient. In several studies, IPC was found to be more effective than IPostC at reducing IRI-induced apoptosis.

The second is ischemic post-conditioning (IPostC) that involves administering bursts of controlled reperfusion before continuous reperfusion inside the recipient after a prolonged period of ischemia to the organ graft [35]. Although the protection mechanism offered by both procedures are nearly similar, the demonstration of the shared mechanism involved in the operation of IPC and IPostC is shown in Figure 3. 

IPostC has an upper hand over IPC due to the no-administration time limitations. The slower reperfusion allows the protective endogenous substance to not flush out abruptly. IPostC induces an increase in antioxidant enzyme expression, a decrease in neutrophil infiltration, and an increase in anti-apoptotic enzyme expression [36]. IPostC has also been shown to alter MPT and up-regulate the behavior of eNOS and inducible nitric oxide synthase (iNOS), which may explain the beneficial effects on microcirculation. Nevertheless, the promising literature of the clinical trials shows no benefits of IPC and IPostC on postoperative liver function tests or long-term mortality and morbidity.

Hypothermic machine perfusion (HMP) is a technique that has come under a lot of attention in the field of liver transplantation, specifically in relation to ECD organs. Machine perfusion (MP) aims to enable ECD organs to be reconditioned, tested for function, and their preservation to be extended. HMP uses a perfusate close that is used in static cold storage (SCS), and the perfusion technique is less expensive and time-consuming than normothermic machine perfusion (NMP). The key drawback of HMP is that it does not allow for the generation of data to determine liver function. Low temperatures have the additional advantage of reduced metabolic activity, followed by increased viscosity as a result, thereby requiring higher perfusion pressure that increases the risk of SEC damage. HMP perfusate oxygenation (HOPE) appears to be the secret to preventing hepatic IRI. HOPE, in comparison to NMP, reduces ROS release during reperfusion. HOPE also reduces inflammatory response pathways and results in a complete restoration of mitochondrial ATP status in just two hours [15]. The second type is normothermic machine perfusion, wherein during the preservation time, NMP uses a blood-based perfusate to perfuse the liver and maintain physiological metabolism. NMP is usually started at the time of organ retrieval and continued for three to 19 hours during organ transportation before transplantation into the recipient. Hepatic IRI is less severe when NMP is used. NMP is thought to aid in the maintenance of a healthy endothelium and the replenishment of hepatic ATP supplies, as seen in a porcine model. NMP affects the expression of genes that are involved in liver regeneration and inflammation control. Moreover, NMP has been shown to help preserve livers for longer periods, with the longest preservation time being close to 19 hours [37]. 

Mitochondria transplant was studied using animal models of liver, lungs, and brain disorders. These were used to evaluate the transplantation of allogenic mitochondria. A model rat with a liver IRI was used as a subject for mitochondrial transplantation in the liver [38]. The levels of ALT, apoptotic markers, and the development of ROS were all reduced after allogeneic liver mitochondria were implanted via intrasplenic injection. Weekly intravenous doses of allogenic liver mitochondria at multiple three-day intervals were used to validate the therapeutic effect in a mouse model of fatty liver. The injection of isolated mitochondria resulted in a substantial decrease in fatty liver endpoints such as ALT levels, according to the findings.

From the above discussion, we interpret that even though there has been much research on I/R damage, the molecular and cellular pathways involved in this process are still unknown and need to be investigated further. Also, the novel strategies mentioned above have a very promising approach in the literature and hence should be taken further into human trials.

Limitation

Owing to the lack of full papers, we were able to include only a small number of studies in this review. The sample articles used for the data in this study mostly include literature reviews and a few randomized control articles. This review excludes studies from the past 10 years.

## Conclusions

Hepatic ischemia/reperfusion injury is a complex pathophysiological event that is responsible for nearly 10% of the primary graft rejection after liver transplantation. Even though there has been much research into I/R damage, the molecular and cellular pathways involved in this process are still unknown and need to be investigated further. The purpose of this review is to highlight the main sequence of incidents entailing mitochondrion processes involved in hepatic IRI following LT and to cover therapeutic strategies targeting or blocking these molecular events to prevent primary rejection. Pharmacological agents could be applied to the donor liver protection solutions to enhance the results of LT. Although animal models have been thoroughly investigated, few human experiments have been performed. And most of these experiments were done in impractical settings with little possibility of translation to clinical use.

This review is written by collecting data from already published studies and covers a wide range of pathophysiological processes leading to IRI. The creation of new mitochondrial drug delivery systems may aid some of these therapeutic targets to impact the mitochondria. Since it's clear that mitochondrion-related processes and targeting therapies demonstrate the potential for better graft survival, the authors of this review recommend further clinical trials of the existing mitochondria targeting therapies and novel techniques to enable the success of the liver graft.
